# Supplementary material for: Complexome profiling of the Chlamydomonas psb28 mutant reveals TEF5 as an early PSII assembly factor
Source: Plant Cell. 2025 Mar 17;37(6):koaf055. doi: 10.1093/plcell/koaf055 (PMC12164590; doi:10.1093/plcell/koaf055)
Supplement: koaf055_Supplementary_Data [file koaf055_supplementary_data.zip › psb28_interactive.zip!/plots/intensity/qualityPlots/peptides/atpF_Cre-1.g2716983.t1.p1.html]

### Cre-1.g2716983.t1.p1

Trivial name: atpF  
SeqCoverage [median]: 71.40  
Mass: 20.06 kDa  
  
 

| Sequence | MissedCleavage | Mass [Da] | wtIntensity | muIntensity | Score |
| --- | --- | --- | --- | --- | --- |
| AFKQAYLYVINK | 1 | 1456.81 | 1474.42 | 0.00 | 43.68 |
| AFKQAYLYVINKIMTR | 2 | 1958.08 | 26224.56 | 11487.64 | 87.99 |
| AIEAEQK | 0 | 787.41 | 455922.51 | 438975.43 | 160.03 |
| AIEAEQKLTAAR | 1 | 1299.71 | 25978.83 | 72533.82 | 134.98 |
| AQEIREEGVLR | 1 | 1298.69 | 596506.44 | 427055.70 | 179.57 |
| ATQEINNVVSQHELR | 0 | 1736.88 | 1068471.81 | 1023089.76 | 226.24 |
| EEGVLR | 0 | 701.37 | 76024.15 | 19651.62 | 166.22 |
| GLDSTYHVVVNNFYVSR | 0 | 1968.97 | 374285.03 | 598752.21 | 235.35 |
| GLDSTYHVVVNNFYVSRFTQF | 1 | 2492.21 | 1355.48 | 0.00 | 45.37 |
| KAQEIREEGVLR | 2 | 1426.79 | 152010.32 | 119712.21 | 140.45 |
| KKAQEIREEGVLR | 3 | 1554.88 | 27813.14 | 59970.19 | 134.56 |
| KNTIVK | 1 | 701.44 | 9552.84 | 0.00 | 99.38 |
| LNKGLDSTYHVVVNNFYVSR | 1 | 2324.19 | 0.00 | 19509.95 | 83.76 |
| LQEFKQETLAFYQQK | 1 | 1899.97 | 359458.03 | 643651.58 | 229.39 |
| NLEEANQR | 0 | 972.46 | 605101.46 | 468430.14 | 230.54 |
| NLSSLLEDR | 0 | 1045.54 | 161567.66 | 321987.69 | 186.78 |
| NLSSLLEDRK | 1 | 1173.64 | 37377.78 | 48410.24 | 113.03 |
| QAYLYVINK | 0 | 1110.61 | 260458.54 | 367949.63 | 235.24 |
| QETLAFYQQK | 0 | 1254.62 | 205185.57 | 103472.39 | 399.41 |
| TQLETAK | 0 | 789.42 | 434100.84 | 474913.03 | 242.52 |
| TQLETAKK | 1 | 917.52 | 57147.35 | 32634.59 | 136.27 |
|  |  |  | 4936016.7 | 5252187.8 |  |
